# A Computational Approach in the Systematic Search of the Interaction Partners of Alternatively Spliced TREM2 Isoforms †

**DOI:** 10.3390/ijms25179667

**Published:** 2024-09-06

**Authors:** Junyi Liang, Aditya Menon, Taylor Tomco, Nisha Bhattarai, Iris Nira Smith, Maria Khrestian, Shane V. Formica, Charis Eng, Matthias Buck, Lynn M. Bekris

**Affiliations:** 1 Genomic Medicine Institute, Cleveland Clinic, Cleveland, OH 44195, USA; liangj3@ccf.org (J.L.); tomcot@ccf.org (T.T.); smithi4@ccf.org (I.N.S.); khrestm@ccf.org (M.K.); formics@ccf.org (S.V.F.);; 2 Department of Biomedical Engineering, Case Western Reserve University, Cleveland, OH 44106, USA; aam218@case.edu; 3 Department of Physiology and Biophysics, Case Western Reserve University, Cleveland, OH 44106, USA; nxb446@case.edu; 4 Cleveland Clinic Lerner College of Medicine, Cleveland, OH 44195, USA; 5 Department of Neurosciences, Lerner Research Institute, Cleveland Clinic, Cleveland, OH 44195, USA

**Keywords:** Alzheimer’s disease, TREM2 isoforms, structural bioinformatics, binder

## Abstract

Alzheimer’s disease is the most common form of dementia, characterized by the pathological accumulation of amyloid-beta (Aβ) plaques and tau neurofibrillary tangles. Triggering receptor expressed on myeloid cells 2 (TREM2) is increasingly recognized as playing a central role in Aβ clearance and microglia activation in AD. The *TREM2* gene transcriptional product is alternatively spliced to produce three different protein isoforms. The canonical TREM2 isoform binds to DAP12 to activate downstream pathways. However, little is known about the function or interaction partners of the alternative TREM2 isoforms. The present study utilized a computational approach in a systematic search for new interaction partners of the TREM2 isoforms by integrating several state-of-the-art structural bioinformatics tools from initial large-scale screening to one-on-one corroborative modeling and eventual all-atom visualization. CD9, a cell surface glycoprotein involved in cell–cell adhesion and migration, was identified as a new interaction partner for two TREM2 isoforms, and CALM, a calcium-binding protein involved in calcium signaling, was identified as an interaction partner for a third TREM2 isoform, highlighting the potential role of cell adhesion and calcium regulation in AD.

## 1. Introduction

Alzheimer’s disease (AD) is a progressive brain disorder that affects a person’s memory, thinking, and behavior [1,2]. It typically has a significant impact on the individual patient and their ability to function independently as well as on their family and caregivers and also poses a significant economic burden on society due to the high cost of care [3,4].

**Figure 1:**
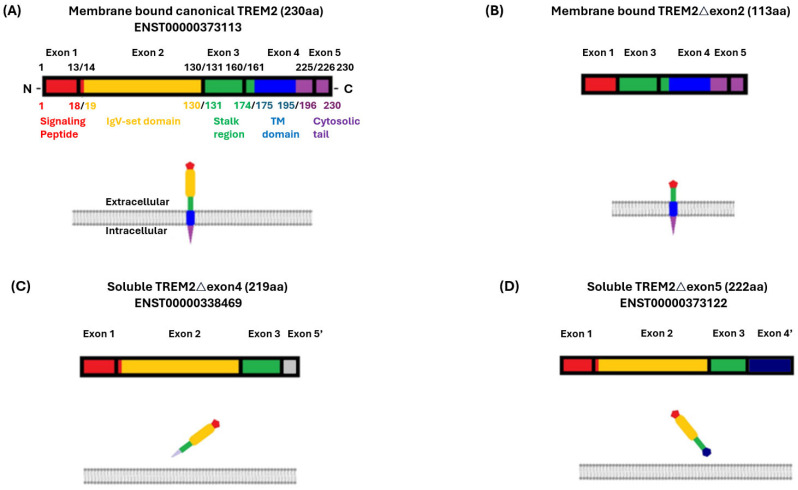
Schematic introduction to the TREM2 isoforms: canonical TREM2 and TREM2 isoforms produced by alternative splicing. (**A**) The primary structure of canonical TREM2, including its composition with respect to the exons and corresponding localization in the cellular setting, and the same depiction for (**B**) TREM2△exon2. (**C**) TREM2△exon4 and (**D**) TREM2△exon5. Red stands for signaling peptide, yellow stands for IgV-set domain, green stands for stalk region, blue stands for transmembrane domain/TM domain, purple stands for cytosolic tail, grey stands for an exon5 altered from the original exon 5, and navy blue stands for an exon 4 altered from the original exon 4.

Triggering receptor expressed on myeloid cells 2 (TREM2) is a protein that is primarily expressed in myeloid cell types in the brain and has emerged as a significant player in AD and other neurodegenerative diseases [5,6,7] as well as a promising therapeutic target [8,9]. *TREM2* RNA undergoes alternative splicing to generate three *TREM2* isoforms in addition to the canonical *TREM2*, specifically producing TREM2△exon2, TREM2△exon4, and TREM2△exon5 [7,10]. Canonical TREM2 is membrane-bound, owing to exon 4, which is home to its transmembrane domain (Figure 1A). TREM2 has a cytoplasmic tail [11,12] that interacts with intracellular signaling molecules and, in turn, transduces signals into the cell [13,14]. TREM2△exon2 is believed to be a membrane protein not unlike canonical TREM2, but it differs from the canonical protein because it lacks the ligand-binding domain encoded by exon 2, while all other exons are intact (Figure 1B) [15,16]. TREM2△exon4 skips exon 4, causing a frameshift and an alternation to exon5 (Figure 1C) [17]. TREM2△exon5 has an untranslated exon 5 region and harbors a changed sequence in exon 4 (Figure 1D) [7,18,19]. TREM2△exon4 and TREM2△exon5 are likely soluble due to the absence of a functional transmembrane domain and are reportedly exported to the extracellular environment (Figure 1C,D) [7].

Canonical TREM2 engages in protein–protein interactions that play an integral role in cell signaling and the function of microglia, the immune cells of the central nervous system, as well as monocytes, dendritic cells, and macrophages in the peripheral system [19]. Canonical TREM2 may interact with lipids and lipoproteins [20], including high-density lipoprotein and low-density lipoprotein [21], as well as apolipoproteins in the extracellular environment, where apolipoprotein (ApoE) plays a critical role in lipid handling and clearance of cellular debris [11,22,23,24]. The direct interaction of the canonical TREM2 ligand-binding region and Aβ42 has also been described [25]. The interaction between canonical TREM2 and DAP12 is known to initiate important immune responses that are crucial for phagocytosis that, in turn, plays a critical role in the clearance of Aβ [26,27,28]. The development of small molecules that stimulate the interaction between canonical TREM2 and DAP12 and downstream signaling is being pursued as a new therapeutic opportunity [29].

In addition, soluble TREM2 generated by alternative splicing is detectable in various biological samples, including plasma and cerebrospinal fluid [30,31,32,33]. Studies have demonstrated its presence in both Alzheimer’s patients and non-patients. In Alzheimer’s patients, both TREM2△exon4 and TREM2△exon5 are less abundant than the canonical TREM2 in the brain [17,34]. In addition to these presumed soluble TREM2 protein isoforms produced by alternative splicing, another soluble TREM2 protein is produced by α-secretase cleavage of the canonical isoform [34,35]. Remarkably, soluble TREM2 levels exhibit distinct patterns in the different stages of AD progression. Cerebrospinal fluid-soluble TREM2 is notably elevated in the early stages of AD, but its levels tend to decrease as AD progresses [30,35,36,37], and is presumed to hold important regulatory functions in the extracellular environment through its interaction with biomolecules in the cerebrospinal fluid [38]. For example, soluble TREM2 is proposed to be neurologically beneficial in the setting of AD due to its ability to bind Aβ and lessen Aβ oligomerization-dependent neuronal damage [39,40]. TREM2 is also reported to be cleavable by γ-secretase, which processes amyloid-beta precursor protein (APP), the precursor to Aβ [41]. However, little is known about alternatively spliced TREM2 isoforms’ function or interaction with other biomolecules.

**Figure 2:**
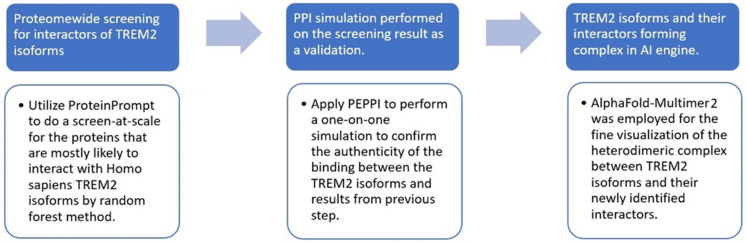
General workflow diagram. In step 1, proteome-wide screening by ProteinPrompt was used to identify the prospective interaction partners for TREM2 isoforms. In step 2, independent modeling by PEPPI was used to validate step 1’s predictions. In step 3, the interaction partners identified in step 1 and validated in step 2 were built into a heterodimeric complex with the corresponding TREM isoform by AlphaFold2 Multimer.

We hypothesized that, in addition to canonical TREM2, alternatively spliced TREM2 isoforms have interaction partners other than previously reported DAP12, Aβ42, or ApoE. This study aimed to implement a systematic computational search for new interaction partners of these TREM2 isoforms by employing several state-of-the-art biocomputational tools in a series of three sequential steps, (1) initial large-scale screening, (2) one-on-one corroborative modeling, and (3) fine structural visualization, as shown in the workflow diagram of Figure 2.

Specifically, this computational approach was designed in order to, firstly, perform large-scale screening upfront in the Homo sapiens proteome to find prospective interaction partners for TREM2 variants utilizing ProteinPrompt (https://proteinformatics.uni-leipzig.de/protein_prompt/, accessed on 2 September 2024), an emerging software particularly adapted at performing protein–protein interaction (PPI) screening at scale [42]. Then, secondly, one-on-one modeling was performed on the top five ProteinPrompt predictions for each TREM2 variant as a validation of the authenticity of the newly identified interaction. For this step we used Pipe for the Extraction of Predicted Protein–protein Interactions, otherwise known as PEPPI (https://zhanggroup.org/PEPPI/, accessed on 2 September 2024), a pipeline designed for predicting PPI [43]. Lastly, AlphaFold2 Multimer is an AI engine pre-trained to build dimeric complexes [41,44,45] and was used to visualize the heterodimer, including the dimer interface, formed by alternatively spliced TREM2 isoforms and their newfound interaction partners. In the end, two new interaction partners for certain alternatively spliced TREM2 isoforms were discovered that highlight the potential role of cell adhesion and calcium regulation in AD.

## 2. Results and Discussion

### 2.1. ProteinPrompt Predictions from the Human Proteome

**Figure 3:**
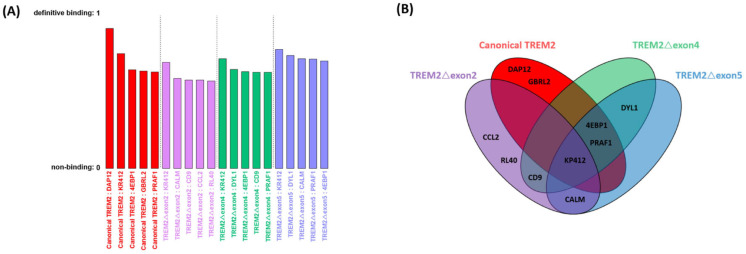
ProteinPrompt’s prediction on the prospective interaction partners of canonical TREM2 and alternatively spliced TREM2 isoforms. (**A**) ProteinPrompt’s top 5 predictions for each of the TREM2 are shown and ranked according to the score in ProteinPrompt, which in turn was calculated by a majority vote in ProteinPrompt’s random forest method. Note that a couple of canonical TREM2’s known interaction partners, such as TDP-43 and ADAM17, were not among the top 5 predictions in ProteinPrompt. (**B**) ProteinPrompt’s prediction for each TREM2 variant’s 5 most likely interaction partners in the proteome of Homo sapiens was obtained with the random forest method. The notable overlaps are the following: KP412 is predicted by ProteinPrompt to interact with all four TREM2 variants, while CALM interacts with TREM2△exon2 and TREM2△exon5; CD9 interacts with TREM2△exon2 and TREM2△exon4; PRAF1 and 4EBP1 interacts with TREM2△exon2, TREM2△exon4, and with TREM2△exon5, (canonical TREM2: red; TREM2△exon2: purple; TREM2△exon4: green; TREM2△exon5: blue).

Utilizing the ProteinPrompt random forest (RF) machine learning algorithm, the five top predictions (candidate interaction partners) were selected from the proteome of Homo sapiens as the most likely interaction partners for canonical TREM2 and alternatively spliced TREM2 isoforms. (1) DAP12, GBRL2, KP412, 4EBP1, and PRAF1 were the top five predictions for canonical TREM2; (2) CCL2, RL40, CALM, KP412, and CD9 were the top five predictions for TREM2△exon2; (3) KP412, CD9, DYL1, PRAF1, and 4EBP1 were the top five predictions for TREM2△exon4; and (4) CALM, KP412, 4EBP1, PRAF1, and DYL1 were TREM2△exon5’s top five predictions, as listed in Figure 3A. There is some overlap worthy of mentioning: for example, CD9 is one of ProteinPrompt’s top five predictions for both TREM2△exon2 and TREM2△exon4, and CALM is among ProteinPrompt’s top five predictions for both TREM2△exon2 and TREM2△exon5 (Figure 3B). Here, the identified top five predictions for each alternatively spliced TREM2 isoform were the starting point for the next step of one-on-one modeling. Note that not all the known interaction partners of the TREM2 variants are in the top five results of ProteinPrompt (e.g., Aβ is not). This might be caused by the built-in filters, which were meant to reduce false positives, inadvertently diminishing the ranking of these true interactions.

### 2.2. PEPPI Validation of Top Five Protein Prompt Predictions

**Figure 4:**
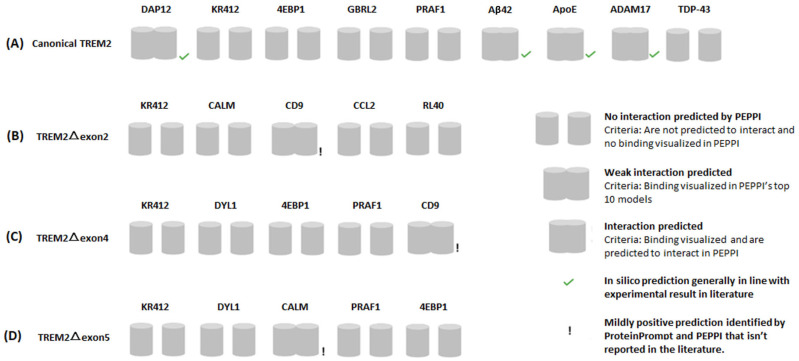
PEPPI modeling results for (**A**) canonical TREM2, (**B**) TREM2△exon2, (**C**) TREM2△exon3, (**D**) TREM2△exon4. A cross-check on ProteinPrompt’s top 5 predictions. PEPPI succeeded in reproducing some known interactions of canonical TREM2, namely, its interaction with Aβ42 and ApoE. Canonical TREM2’s interaction with ADAM17 as well as DAP12 was reproduced by PEPPI. We note, however, that PEPPI did not reproduce the interaction between canonical TREM2 and TDP-43. CD9, one of ProteinPrompt’s top predictions for TREM2△exon2, was shown to bind with this isoform by PEPPI. CD9 was predicted to bind TREM2△exon4, and the interaction with CALM, another top prediction for TREM2△exon5, was also shown by PEPPI. However, similar to the canonical TREM2, PEPPI provided no confirmation of ProteinPrompt’s other top predictions for the splice variants.

The PEPPI analyses reproduced some known interactions of canonical TREM2, namely, its interaction with Aβ42 and ApoE. DAP12, one of ProteinPrompt’s top five likely interaction partners for canonical TREM2, is known to interact with canonical TREM2. Indeed, DAP12 and canonical TREM2 binding was visualized by PEPPI (Figure 4A).

PEPPI verified the validity of CD9 (cluster of differentiation 9; Uniprot ID: P21926) as an interaction partner, as binding was visible in the modeling of both TREM2△exon2:CD9 and TREM2△exon4:CD9 (Figure 4B,C).

A look into the biology of CD9 is intriguing because it is involved in cell adhesion and membrane organization. It is expressed in microglia, in platelets and in immune cells such as T cells [46,47]. The implication for AD is that TREM2-expressing microglia are involved in Aβ clearance through phagocytosis. CD9, through its involvement in membrane organization and cell adhesion, could potentially influence the interaction between microglia and Aβ plaques via its interaction with TREM2. CD9 has also been implicated in cell migration [48,49]. Moreover, TREM2 has been proposed to mediate microglial migration and interactions with other cell types [50]. Taken together, this suggests that an interaction between CD9 and TREM2 may influence microglial migration and positioning and their interactions with other cells in the brain. It is also worth mentioning that the interaction of CD9 and canonical TREM2, as evidenced by their colocalization, has already been observed using immunofluorescence staining in lipid-associated macrophages from mice on a high-fat diet [51] (CD9 is ranked as sixth among the interaction partners for canonical TREM2 in ProteinPrompt).

CALM (calmodulin 2; Uniprot ID: P62158) binding with TREM2△exon5 was replicated by PEPPI (Figure 4D). Interestingly, in AD, calcium dysregulation is observed, leading to altered calcium homeostasis in neurons and glial cells [52]. It is plausible that an interaction with TREM2 could potentially modulate CALM’s function in microglia. Also, both CALM and TREM2 have been implicated in synaptic function and plasticity. CALM interacts with proteins involved in neurotransmitter release and synaptic plasticity [53,54]. Disruptions in synaptic function and plasticity are early pathological features of AD [55,56,57]. TREM2 has been associated with synaptic pruning and modulating synaptic connectivity [58,59,60]. Furthermore, it has been suggested that TREM2 contains a CALM-binding motif [61]. Taken together, this suggests that an interaction between CALM and TREM2 may influence synaptic function in the brain.

### 2.3. AlphaFold2 Visualization

**Figure 5:**
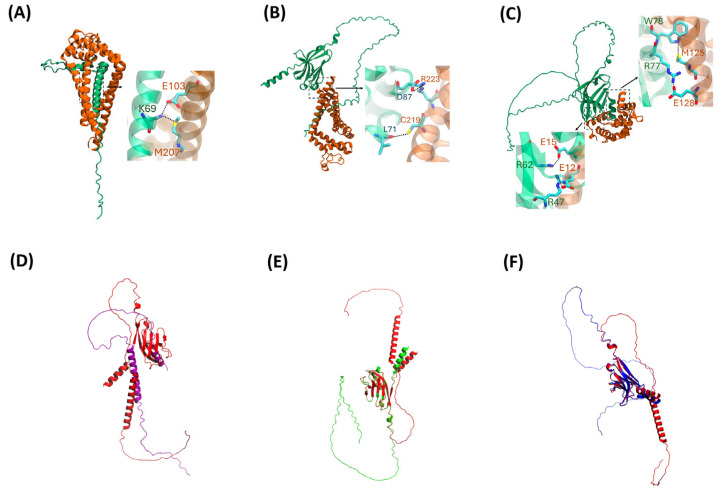
AlphaFold2 Multimer visualization of the dimeric complexes formed by TREM2 alternatively spliced isoforms and the newfound interaction partners with part of the interacting interface zoomed in: (**A**) TREM2△exon2 (dark green) and CD9 (orange), (**B**) TREM2△exon4 (dark green) and CD9 (orange), (**C**) TREM2△exon5 (dark green) and CALM (orange)—and structural superposition between canonical TREM2 (red) and the isoforms—(**D**) between canonical TREM2 and TREM2△exon2, (**E**) between canonical TREM2 and TREM2△exon4, (**F**) between canonical TREM2 and TREM2△exon5.

ProteinPrompt identified the likely interaction partners, and PEPPI validated some of these putative interaction partners. However, neither method offers any molecular details regarding the manner of the interaction. AlphaFold2 Multimer predicted structures for the three computationally mined novel interactions identified by ProteinPrompt and validated by PEPPI (Figure 5). The heterodimeric complexes formed by TREM2△exon2 and CD9 (Figure 5A), TREM2△exon4 and CD9 (Figure 5B), and TREM2△exon5 and CALM (Figure 5C) indicate distinct structural interactions with the alternatively spliced TREM2 isoforms. Notably, each TREM2 isoform was predicted to interact differently with its newfound interaction partner.

In AlphaFold2 Multimer’s top model, CD9 binds to the stalk and TM regions of TREM2△exon2, the latter of which is encoded by exon 4. Therefore, in AlphaFold2 Multimer’s TREM2Δexon2:CD9 model, the TM region is conducive to its interaction with CD9. In AlphaFold2 Multimer’s heterodimeric complex TREM2△exon4:CD9, CD9 binds to TREM2△exon4 in its immunoglobulin domain (Ig domain), a domain known to mediate PPI important for immune functions [22,25]. TREM2’s Ig domain is encoded by exon 2 and also binds with well-known ligands such as Aβ42 and ApoE. It is plausible that TREM2△exon4’s binding with CD9 and its binding to known ligands may be mutually exclusive due to the overlap of the binding regions.

It should be pointed out that CD9 binds to TREM2△exon2’s stalk and TM region but not TREM2△exon4’s stalk region in the AlphaFold2 complexes, despite both isoforms sharing exon 3. This could be related to the major difference in solubility between TREM2△exon2 and TREM2△exon4, as the former is membrane-bound and the latter is soluble. By contrast, CALM also binds to TREM2△exon5’s immunoglobulin domain, which may compete with the binding of natural ligands. The superposition between predicted canonical TREM2 and its alternatively spliced isoforms structures (Figure 5D–F) also shows a correlated and yet distinct conformational posture, underscoring the structural differences leading to CD9 and CALM to bind to specific regions on each isoform in the AlphaFold2 models.

The TREM2 isoforms:CD9 complexes, as shown in Figure 5A,B, could potentially impact processes such as immune surveillance in AD. An interaction between CALM and TREM2 isoform and the resulting complex (Figure 5C) may influence synaptic processes, potentially impacting synaptic dysfunction through calcium signaling in AD. The specific interacting residues in each of the AlphaFold2 Multimer’s three complexes are listed in Table 1. However, it should be noted that AlphaFold2 results come with the caveat that the pLDDTs of the interfaces of the three predicted complexes are all less than 70%, indicating that AlphaFold2 is only mildly confident about the accuracy of the interfaces modeled. Nevertheless, AlphaFold2 predictions for relatively small interfaces, such as those between transmembrane helices that form (hetero)dimers in type-I receptors, were at 40–60% and were found to be remarkably accurate in one recent study [62].

## 3. Materials and Methods

### 3.1. Protein Prompt

ProteinPrompt is a web server that utilizes machine learning algorithms to calculate protein–protein interactions [42]. The platform offers three distinct computational methods—a random forest (RF), a graph neural network (GNN), and a consensus method (CM)—for the determination of top predictions. This software was used to scan Homo sapiens proteome libaries in order to identify prospective binders for canonical TREM2 (ENST00000373113; Uniprot ID: Q9NZC2), where all three methods were used one-by-one for TREM2△exon2, TREM2△exon4 (ENST00000338469), and TREM2△exon5 (ENST00000373122). The graph neural network and consensus methods returned an overabundance of likely binders, owing to score saturation. In preparation for the next step, candidate binders for each TREM2 family member were obtained utilizing random forest top 5 predictions. This online platform can be accessed here: https://proteinformatics.uni-leipzig.de/protein_prompt/, accessed on 2 September 2024.

### 3.2. Pipe for the Extraction of Predicted Protein–Protein Interactions (PEPPI)

PEPPI is a software program operated by Michigan University for predicting the likelihood that a given pair of polypeptide chains interact directly. This tool integrates structural similarity, sequence similarity, functional association data, and machine learning-based classification [43]. The potential interactions between canonical TREM2 and its top 5 candidate binders obtained in ProteinPrompt RF were simulated by PEPPI, one pair at a time. The same calculations were performed with TREM2△exon2, TREM2△exon4, and TREM2△exon5. PEPPI can be accessed online via this link: https://zhanggroup.org/PEPPI/, accessed on 2 September 2024. The inputs for PEPPI are the primary structures of the two polypeptide chains; in our work, the inputs are the sequence of one of the TREM2 isoforms and the sequence of one of its top 5 candidate binders as predicted by ProteinPrompt. The coordinate files for the 10 models deemed by PEPPI to be most probable were the output.

### 3.3. AlphaFold2 Multimer

AlphaFold2 Multimer v3 is an extension of AlphaFold2, a computational structural biology program developed by Google’s artificial intelligence arm Deepmind and a newer iteration of AlphaFold [41]. AlphaFold2 Multimer was used to model the conformation of protein complexes. UCSF chimera, a molecular graphics system, was used as the environment to access ColabFold, a faster version of AlphaFold2 for complex building [63,64]. The input to AlphaFold2 Multimer is the primary structure of the two polypeptide chains. Thus, in our work, the inputs were the sequences of TREM2△exon2 and CD9, the sequences of TREM2△exon4 and CD9, and the sequences of TREM2△exon5 and CALM. The most likely complex model’s coordinate file for each pair is the output.

## 4. Conclusions

In summary, we carried out a proteome-wide search of alternatively spliced TREM2 isoform interaction partners in silico. The investigation involved both large-scale screening of the proteomes of Homo sapiens as well as conformational one-on-one modeling by state-of-the-art in silico tools. CD9, a cell surface glycoprotein belonging to the tetraspanin family, was identified as a novel interaction partner for TREM2△exon2 and TREM2△exon4. CD9 is known as a key player in cell adhesion and is found on the surface of various cell types that are an integral part of the immune system, such as white blood cells and platelets. The newly discovered interaction between CD9 and TREM2 isoforms raises the prospect that TREM2 isoforms could be playing a role in cell–cell interaction within the same cell type or between different types of cells relevant to brain immunomodulation and, by extension, to AD.

CALM, a calcium-binding protein, is a novel interaction partner for TREM2△exon5 identified in this work. Calcium is a key element involved in maintaining neuronal health and the interaction between CALM and TREM2 isoform suggests that TREM2 may also influence calcium dynamics.

All in all, the protein interaction partners determined in this computational study for alternatively spliced TREM2 isoforms suggest possible new functions of TREM2 isoforms with respect to Alzheimer’s disease, including the role of TREM2 isoform in cell adhesion and calcium signaling. Remarkably, this in silico approach is an experiment in accelerating the study of the protein-protein interactions of a key protein of urgent biological interest, compared with typically slower biochemical approaches. It will be important to conduct a comprehensive biophysical characterization of the interaction between TREM2 isoforms and their newfound binders by biocompatible imaging approaches, such as fluorescent protein technology [62,65,66,67,68,69], to understand the manner these predicted binders interact with TREM2 isoforms biologically.

## Figures and Tables

**Table 1:** The interacting interfaces in AlphaFold2 Multimer’s three heterodimeric complexes.

Interacting Residues		
TREM2△exon2:CD9	TREM△exon4:CD9	TREM2△exon5:CALM
S57:T58	L71:C209; L71:M215	R47:E12
K69:M207	D87:R223	R62:E15; R62:A11
K69:E103	L89:I216	H67:E15
I8:R36	L75:R222	R77:E128
D14:K42	F74:C219	R98:E121
I68:A24; I68:V28		H114:E8
F17:A72; L62:I102		W78:K116; W78:M125; W78:L117
L59:A106		W70:F93; F74:M110
		M77:L69; L75:L113

## Data Availability

The data presented in this study are available upon reasonable request to the corresponding authors.
